# Impact of canine epilepsy on judgement and attention biases

**DOI:** 10.1038/s41598-020-74777-4

**Published:** 2020-10-20

**Authors:** Sarah L. Hobbs, Tsz Hong Law, Holger A. Volk, Chantal Younis, Rachel A. Casey, Rowena M. A. Packer

**Affiliations:** 1 Royal Veterinary College, Hawkshead Lane, Hatfield, AL9 7TA Hertfordshire UK; 2 Department of Small Animal Medicine and Surgery, University of Veterinary Medicine Hannover, Bünteweg, 30559 Hannover, Germany; 3 Dogs Trust, 17 Wakley Street, The Angel, London, EC1V 7RQ UK

**Keywords:** Animal behaviour, Neurological disorders

## Abstract

Idiopathic epilepsy (IE) is the most common chronic neurological condition in dogs, characterised by recurrent seizure activity and associated with negative behavioural and cognitive changes. We hypothesised that IE would negatively impact putative affective state, with dogs with IE exhibiting a more pessimistic judgement bias and more negative attention bias than controls. Dogs were tested in a previously-validated spatial judgement bias task, and a novel auditory attention bias task testing attention to sounds with different valence or salience (neutral, novel pre-habituated, threatening). Sixty-eight dogs (IE = 33, Control = 35) were tested, of which n = 37 acquired the spatial discrimination and responses to judgement bias probes were tested (IE = 19, Control = 18), and n = 36 were tested for responses to sounds (IE = 20, Control = 16). Study groups did not significantly differ by age, sex, breed or neuter-status (p > 0.05). Main effects of study group were not significant in judgement bias (F_1,102_ = 0.20, p = 0.658) or attention bias tasks (F_3,102_ = 1.64, p = 0.184). In contrast with our hypotheses, there was no evidence that IE altered cognitive biases in this study population; however, dogs with IE were significantly more likely to be unable to learn the spatial discrimination task (p = 0.019), which may reflect IE-related cognitive deficits. Developing methods to test affective state without excluding cognitively impaired individuals is a future challenge for animal welfare science.

## Introduction

Idiopathic epilepsy (IE) is the most common chronic neurological condition in dogs, with an estimated prevalence of 0.6–0.75%^1,2^. This represents ~ 71,000 of the 11.5 million pet dogs in the UK alone^3^. Epilepsy is characterised by recurring seizure activity, which in practice represents an individual experiencing at least two unprovoked seizures at least 24 h apart^4^. The most common type of epilepsy in dogs, IE, is epilepsy with a presumed genetic origin, where the cause cannot be explained by any identifiable abnormalities or underlying diseases^5,6^. Age of onset is most commonly between 6 months and 6 years of age^7^ and the condition is usually lifelong; in many cases requiring daily medication^8^ and significantly reducing lifespan^9^.

IE negatively influences the quality of life (QOL) of affected dogs in a multitude of ways^10^. Seizures are sometimes preceded by a prodromal (pre-seizure) phase which can include signs of distress including increased fearfulness and restlessness^11^. Seizures also induce a stress response, with cortisol levels significantly increased 40 min after a seizure compared with a time-matched point on a non-seizure day^12^. Seizures are commonly followed by a post-ictal phase, often characterised by reduced mentation, ataxia and disorientation^10^. Correspondingly, owners of dogs with higher seizure frequencies have reported in survey-based studies that their dogs have a lower QOL than owners of dogs with lower seizure frequencies^13^. In addition to seizure activity, therapeutic attempts to reduce their frequency and/or severity may further impair QOL due to common aversive side-effects of anti-seizure drugs (ASDs); indeed, the side effects ataxia and lethargy are associated with a reduced owner-reported QOL^13^. IE is also associated with negative perturbations in behaviour and cognition, including decreased trainability^14^, signs of cognitive impairment^15,16^, increased attention seeking^17^, aggression^18^ and ADHD-like behaviours e.g. behaviours associated with high excitement and/or poor impulse control^19,20^.

Anxiety is an emotional response to a situation or stimulus which can be perceived as threatening^21^, and is a common reason for dogs to show behaviours that owners find a problem^22^. In humans, a comorbid relationship between epilepsy and anxiety is well established^23^ with a hypothesised pathophysiology related to abnormal functioning γ-Aminobutyric acid (GABA) receptors^24^. People with epilepsy are more likely to report anxiety than those without epilepsy^25^, or patients with other chronic medical issues^23^. Several forms of anxiety are seen in epilepsy patients, including ictal anxiety (e.g. focal seizures with amygdala involvement inducing patients to experience fear/panic during the seizure), postictal anxiety (e.g. during recovery from a seizure, often associated with disorientation) and interictal anxiety (e.g. between seizures, as a comorbidity, as a seizure phobia, as a side effect of ASDs)^23^. Evidence on the link between seizure frequency and the severity of anxiety is mixed^26,27^. In recent survey-based studies, owners of dogs with epilepsy have reported increases in fear and anxiety following the onset of epilepsy in their dog^18^. Increased anxiety was observed in drug naïve individuals and those receiving ASDs. In human epilepsy patients, anxiety has a significant effect on health-related QOL, to a greater degree than seizure frequency, severity or chronicity^23,28^. The impact of epilepsy and its comorbidities on affective state are yet to be explored in the dog in an empirical manner, with studies to date relying on survey-based owner reports.

Affective state can influence the judgement of ambiguous stimuli; and a negative affective state such as anxiety is associated with a more negative or pessimistic interpretation of ambiguous stimuli^29–33^. Despite inherent challenges^34^, indicators of presumed affective state based on relative judgement bias have been investigated in non-human animals^31,35^. Judgement bias tasks broadly measure a baseline response to stimuli of different valence or value and then record response to an intermediate or ambiguous stimuli as an indicator of affective state^36^. The value of this type of approach over physiological indicators of ‘stress’ or arousal is the ability to detect the valence of affective state^31^. The judgement bias paradigm has been used to provide evidence of long-term impacts on affective state in dogs, including neurological disorders (e.g. syringomyelia in Cavalier King Charles Spaniels^37^) and behavioural disorders (e.g. separation-related behaviours^38^) with affected dogs exhibiting more negative judgements of the ambiguous stimuli than controls.

More recently, further cognitive tasks have been developed to detect affective state in animals, based on extensively used human techniques^39,40^. Attention modulated by the observer’s affective state is typically referred to as “attention bias” in animal welfare science, with attention bias tasks aiming to quantify attention allocation to experimental presentations of stimuli^40^. Attention bias tasks are based on the principle that anxious individuals are more likely to direct attention to potentially threatening, or even innocuous, stimuli^21,30,41^. Attention bias tasks have been tested in a variety of species, and recently validated in sheep^42,43^ and beef cattle^44^ by pharmacologically inducing anxiolytic, anxiogenic, and controlled states prior to testing and quantifying behavioural measures such as attention paid to the threat and latency to feed. Some of these studies have demonstrated, as predicted, that more anxious individuals pay more attention to threatening environmental stimuli (e.g. in studies involving sheep this is commonly a dog), compared to non-anxious individuals. However, it should be noted that many attention bias paradigms are in their infancy, and results to date are mixed e.g. where affect manipulations have led to null results (e.g. sheep administered synthetic stress hormones did not show different levels of attention towards a dog compared with controls)^45^, or results were opposite to the hypothesis and negative affect manipulations led to less attention towards threats (e.g. sheep chronically stressed by lying deprivation showed reduced attention to a dog)^46^. To date, the authors are not aware of attention bias paradigms that have been developed with dogs as the test subject rather than the threatening stimuli, but such methods potentially offer advantages over judgement bias tasks, which often involve lengthy training periods and lead to attrition of subjects^40^. Attention bias has already been found to be influenced by epilepsy in human studies. In a study of people with temporal lobe epilepsy (TLE), individuals who experienced seizures precipitated (‘triggered’) by emotional distress were tested in two attention bias paradigms: an emotional Stroop test and a dot detection task, and their performance compared to individuals with TLE with non-emotional seizure triggers. Individuals with emotional seizure triggers exhibited an attentional bias towards threatening stimuli compared to neutral stimuli, as compared to those with non-emotional seizure triggers^47^. The authors further hypothesized that attentional biases related to threat in patients with TLE may exacerbate disease, by sustaining emotional vulnerability and seizure occurrence^47^.

Given the potential negative impact of IE and its comorbidities on affective state, this study aimed to use an existing judgement bias task and a novel attention bias task to investigate its impact on affective state in dogs when compared to healthy controls. We hypothesised that IE would negatively impact putative affective state, with dogs with IE exhibiting a more pessimistic judgement bias and more negative attention bias than controls.

## Methods

### Subjects and recruitment

A cohort of dogs diagnosed with IE, and an age, breed and sex matched cohort of healthy control dogs were recruited onto the study via social media, veterinary practices and attending canine health related events. Two breeds were recruited to the study, Border Collies and Labradors Retrievers, chosen due to their popularity in the UK, and predisposition to (often drug-resistant) epilepsy^48^. Eligibility was screened using an online sign-up questionnaire. All dogs (from case and control groups) were eligible if they were over one year of age, had no health problems affecting vision, hearing or mobility, and did not show marked negative behavioural responses to unfamiliar people or around food bowls, as these factors may influence ability to perform the tasks included in the test. To be included in the IE groups, dogs must have been clinically diagnosed with IE to at least a tier 1 confidence level^49^. Control dogs were eligible if free of neurological conditions, and were recruited based on their match with a member of the IE group, on age (within 2 years due to the effect of judgement bias^50^), sex and breed.

### Behaviour tasks

#### General testing procedure

Each dog took part in the tasks individually in a standardised room at the Royal Veterinary College, London, away from a clinical setting. Owners were not present during testing to promote consistency between dogs; however, all dogs met the experimenters with their owners prior to the tasks, in a relaxed manner, for at least 10–15-min to habituate to them. Once separated from their owners, all dogs were given a 10–15-min habituation period in the study room with the experimenters before the behaviour tasks began. Throughout this habituation period and during all behavioural tasks, each dog was monitored for potential signs of stress, anxiety and frustration caused by the setting or the tasks (potential behaviours listed in Supplementary File 1). If a dog was distressed at any point during the study, the tasks were abandoned, and the dog returned to their owner. Dogs were identified as not motivated to participate if they could not engage with the behaviour tasks but did not show signs of being anxious. Testing was ceased for any dogs that appeared fatigued by the task (e.g. slowed walking speed, increased lying behaviour, struggling to stand).

#### Task 1: Judgement bias task

**Figure 1:**
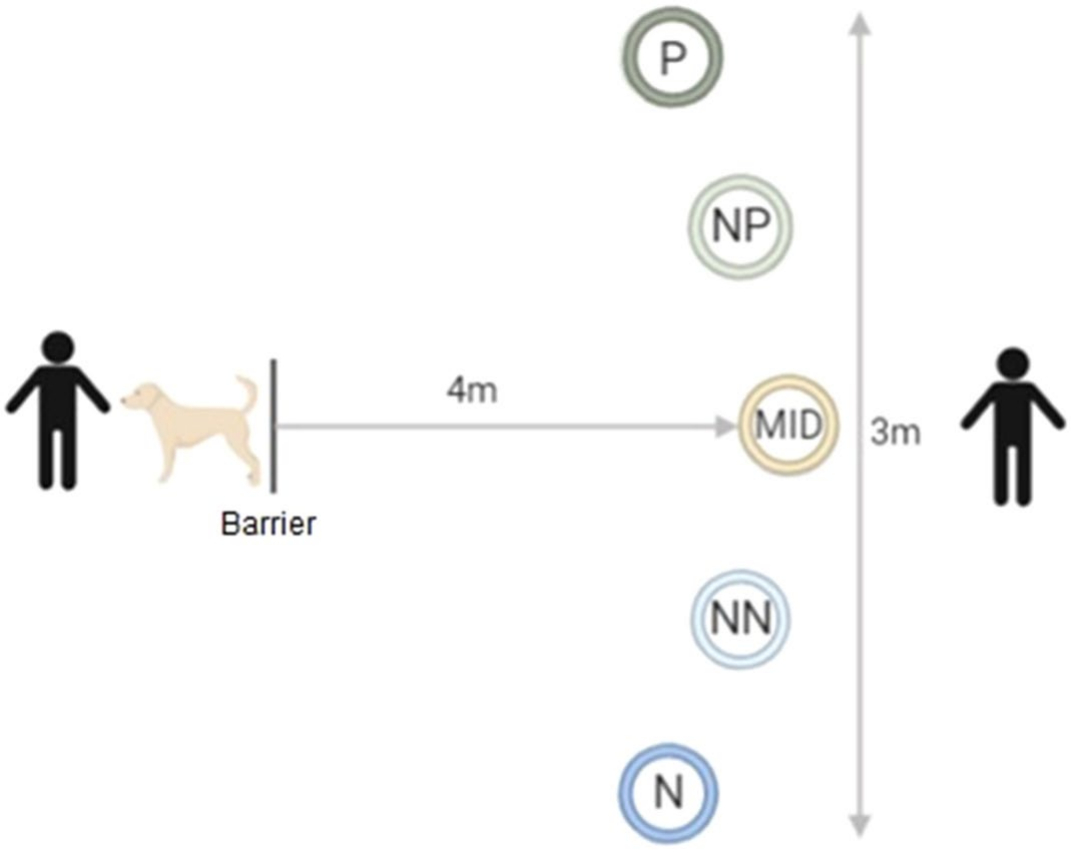
Schematic of the judgement bias task setup. The distance from the starting location to each bowl location was 4 m, with each of the five target locations equidistant from each other over a total of 3 m.

The methodology described by Mendl et al.^38^ was used for the judgement bias task, with the layout of the testing arena shown in Fig. 1. Dogs were kept behind a barrier on a long line lead with no pressure placed on it by experimenter 1 whilst experimenter 2 placed the bowl in one of the five locations. During the training phase of the task, the bowl was baited with one approximately 0.5 cm^3^ cube of cheese (or if an individual dog was unable to have cheese, a suitable alternative food that the dog was motivated to seek out) and placed in the positive (P) location, or empty and placed in the negative (N) location. The P location was either presented on the dog’s left or right hand side, the side was pseudo-randomly allocated between dogs to ensure that 50% of the dogs were presented with the P on each side. Each dog’s matched control was presented with the P on the same side. Trials were presented in the same order for each dog: the first two were baited (P) followed by two un-baited (N) trials, the following 46 trials were pseudo-randomised (using randomizer.org) so that there were no more than two of either P or N were presented consecutively. The same bowl was used throughout testing in both locations to limit olfactory cues. On placement of the bowl, experimenter 2 stepped back from the bowl behing the MID position and the dog was released from behind the barrier still on a long line. Latency (s), measured live with a stopwatch, was recorded for the time it took for the dog to travel from the starting point at the barrier to reaching the bowl, with a maximum of 30 s allowed before being called back behind the barrier to begin the next trial. Dogs were classified as having learnt the spatial discrimination between P and N when they were approaching P faster than N by at least 0.5 s for eight (four P and four N) trials consecutively (a criterion set by Burman and colleagues^34^). Dogs were required to complete a minimum of 16 trials and had a maximum of 50 trials to learn the discrimination.

On reaching criterion for learning the discrimination, dogs moved immediately onto the test phase. During the test phase the same experimental protocol was used, but with the addition of presentation of ambiguous ‘probes’ at intervals. These were each presented only once to avoid learning effects, and were presented in the same order (MID, NP, NN) separated by four pseudo-randomised trials (two P and two N). The task ended with a final four two P and two N trials in a randomised order. The maximum number of trials for the entire task was 65. Where dogs reached 50 trials without reaching criterion on learning the discrimination, the task was ended and the test phase not undertaken. Following the probe trials, a further trial involving the placement of an empty bowl in the positive location was conducted, to check that dogs ran just as fast to this empty bowl as to the usual baited bowl in this location (and were thus not relying on odour cues to detected whether the bowl was baited).

During the judgement bias task, experimenter 1 could gently encourage the dog to leave the starting point verbally a maximum of two times. During the first ten trials dogs could be shown the target bowls if they were not orientating towards them: any trials where this happened were recorded at the maximum latency of 30 s. This protocol was followed equally for the P and N bowl locations. If dogs then continued to not approach the bowls within 30 s for trials 11–16 (when no direction to the was given bowls by the experimenters), the task was abandoned. The testing arena was set up within a larger room and allowed dogs to leave if they chose to. If a dog did not move from behind the barrier, or chose to leave the testing arena at any point of the task, the trial was counted as a ‘no-go’ and recorded as a maximum latency (30 s). If five no-go trials occurred consecutively, the task was abandoned for that dog.

#### Task 2: Attention bias task

Following the judgement bias task, each dog took part in the novel attention bias task. This test aimed to measure study dogs’ attention between a food reward and several, differently valenced sound stimuli. For this test the same room set up was used, and experimenters assumed the same roles. The attention bias task comprised of 29 trials, in which the bowl was always placed in location P (continuing with the rewarded (P) side each dog had been trained on in the judgement bias task). Latency to reach the bowl (P) was the outcome measure of this task. The bowl was always baited and the dog had 30 s to approach and reach the bowl from the starting location. On every third trial, one of three noises was played 0.5 s after the dog had left the start position towards the target bowl. Each sound lasted 2 s and was played at the same volume. Piloting was conducted during test development on dogs not included in this study, to identify a sound volume that was not so loud as to induce startle behaviour (e.g. rapid movement of the head, stepping backwards, taking time to recover post-exposure) but dogs showed attention towards (e.g. ear movement orienting towards sound). Subsequently, no dogs were found to exhibit a startle response to the noises in the testing phase. The speaker was placed on a table 2 m behind the MID bowl. This location was selected so that the sounds came from the direction the dog was travelling towards. This location avoided dogs running away from an anxiogenic sound coming from behind them, potentially leading to a reduced latency to reach the bowl. The sound source was hidden by the experimenter standing behind the MID bowl.

The three noises were chosen due to their assumed different emotional salience; a neutral sound (bubbling water), a novel sound (computerised musical notes), and a negatively valenced sound (dog barking defensively). The negatively valenced sound (dog bark defensively) was chosen as exposure to ‘alarm’ (defensive) barks from unfamiliar dogs towards a stranger have been demonstrated to induce a greater response (including more frequent barking) in dogs exposed to them, than barks from a dog left alone^51^. The sound was opportunistically recorded by one of the authors (SLH) from their own dog barking defensively in response to a stimulus they found aversive. Each sound was played three times and presented in a pseudo-randomised order (so that none were presented twice in a row), and the order of presentation was kept the same for each dog. The novel sound was used to test a noise that all study dogs would never have been exposed to, and hence have no prior learning about before testing started. The sound was not intended to be novel at point of exposure, and dogs were habituated to the sound prior to starting the attention bias test. During the habituation process, each dog was monitored for their responses to the sound. If dogs exhibited any signs of distress or sensitisation (Supplementary Table S1), the sound was stopped and the attention bias task was not undertaken.

Dogs that did not meet the learning criterion for the judgement bias task, or who lacked motivation during the training or testing phases of this task were included in the sample of dogs tested for attention bias. For these dogs, the P bowl location was baited five times before moving straight into the attention bias task trials to reinforce this location. Dogs that did not complete the judgement bias task due to distress or physical problems were not tested in the attention bias task on ethical grounds.

Ethics approval was gained from the RVC Ethics and Welfare Committee (Ref URN 2017 1743-2). All methods were performed in accordance with the relevant guidelines and regulations. All owners were provided with a study information sheet and had the study fully explained to them by an investigator. All owners provided written informed consent for their dog’s involvement in the study.

### Statistical analysis

Data were analysed using IBM SPSS Version 26. Data were checked for assumptions of parametric tests including Shapiro–Wilk tests of normality and Levene’s tests of homogeneity of variance. Results are presented as mean ± SD for normally distributed variables and median (interquartile range [IQR]) for non-normally distributed variables. For all tests p < 0.05 was considered significant.

For the cognitive bias task, although running latencies (s) to probe locations are often adjusted, as groups were recruited to be matched by breed, age, and sex, and the arena size for testing was standardised across all owners, the raw latencies were analysed without adjustment, as well as adjusted scores as outlined in Mendl et al. (2010): (mean latency to probe location [i.e. NN, MID, NP]—mean latency to positive location)/(mean latency to negative location-mean latency to positive location)) × 100. This adjustment results in the latencies to reach ambiguous bowls locations being expressed as a percentage of the difference between each animal’s mean latencies to reach the bowls positioned at P and N^38^. Differences in latency to reach each bowl location and in response to each sound were tested at the univariate level using the Freidman’s test with pairwise Dunn-Bonferroni post-hoc tests.

Due to the poor performance of some dogs in the training phase of the cognitive bias task, not all dogs were tested for responses to the probe locations, and thus not all dogs retained a matched pair for those data. As such, to maximise sample size, all dogs who reached this stage were included in analyses and signalment was included in statistical modelling. Mean latency to the P and N bowl was taken from the last three P’s or N’s from the training phase and all P’s or N’s in the test phase, respectively.

General linear mixed models^52^ (LMMs) were constructed with unadjusted or adjusted latency as the outcome variable, testing for the effects of study group (IE vs. control), ambiguous bowl position (NP, MID, NN), and an interaction between study group*bowl position as fixed effects, while accounting for the repeated effect of Dog ID as a random effect. Additionally, the potentially confounding variables breed, sex and age were included as fixed effects.

For the attention bias task, latency to reach P (s) was used as the outcome variable in an LMM, with the effects of study group and sound type (and their interaction) tested as a fixed effects while accounting for the repeated effect of Dog ID as a random effect, and the potentially confounding variables breed, sex and age were included as fixed effects.

## Results

### Demographics

A total of 68 dogs were recruited and included in behaviour testing; n = 33 dogs with IE and n = 35 controls. These dogs constituted 33 matched pairs, with an additional two controls due to drop out of two dogs with IE before behavioural tasks took place. Demographic details are reported in Table 1; there was no difference in breed distribution (*X*^2^ = 0.08, df = 1, p = 0.772), age (t = − 0.28, df = 66, p = 0.777), weight (t = -0.18, df = 66, p = 0.857), sex (*X*^2^ = 0.00, df = 1, p = 0.994) or neuter status (*X*^2^ = 3.34, df = 1, p = 0.068) between IE and control groups.

**Table 1:** Demographic features of the study population and sub-populations.

Signalment	Population					
	Overall		Judgement bias		Attention bias	
	Epilepsy	Control	Epilepsy	Control	Epilepsy	Control
N	33	35	19	18	20	16
Breed (n) (BC/LR)	20/13	20/15	10/9	10/8	12/8	11/5
Age—months (median [IQR])	68.0 [43.0–111.5]	73.0 [43.0–114.0]	60.0 [43.0–103.0]	72.0 [45.3–108.3]	56.5 [43.5–86.3]	74.0 [44.0–114.0]
Weight (kg) (median [IQR])	24.0 [18.7–29.5]	23.0 [19.0–30.0]	25.0 [18.4–30.0]	23.0 [18.6–29.5]	24.5 [18.6–28.8]	21.0 [16.1–30.0]
Sex (n) (M/F)	16/17	17/18	9/10	7/11	8/12	9/7
Neutered (n) (E/N)	8/25	18/17	4/15	9/9	5/15	6/10

*BC* border collie, *LR* labrador retriever, *IQR* Interquartile range, *M* male, *F* female, *E* entire, *N* neutered.

### Epilepsy characteristics

Of the dogs with IE, all had reached IVETF tier I diagnostic certainty, with 45% (n = 15) having undergone MRI to further confirm their diagnosis. Twenty-two dogs (67%) were treated with at least one ASD (n = 11 no ASDs, n = 10 one ASD, n = 8 two ASDs, n = 4 three ASDs). The most commonly used medication was phenobarbital (n = 18), followed by levetiracetam (n = 10), imepitoin (n = 4), potassium bromide (n = 4) and gabapentin (n = 1). Five dogs were supplemented with oils containing medium-chain triglycerides (pure MCT and/or coconut oil), and two with cannabidiol (CBD) oil. Nearly two thirds of dogs had experienced cluster seizures (more than one seizure within 24 h; 64%, n = 21) and nearly one third had experienced status epilepticus (a seizure lasting over 5 min; 27.3%, n = 9). Dogs had experienced a mean of 3.79 ± 3.62 seizures in the past 3 months. Seven dogs (four Labrador Retrievers and three Border Collies) had experienced no seizures in the past 3 months. Of those ASD-treated dogs, the majority (85.0%, n = 17) had experienced a ≥ 50% reduction in seizure frequency following the start of their current ASD regimen.

### Judgement bias

**Figure 2:**
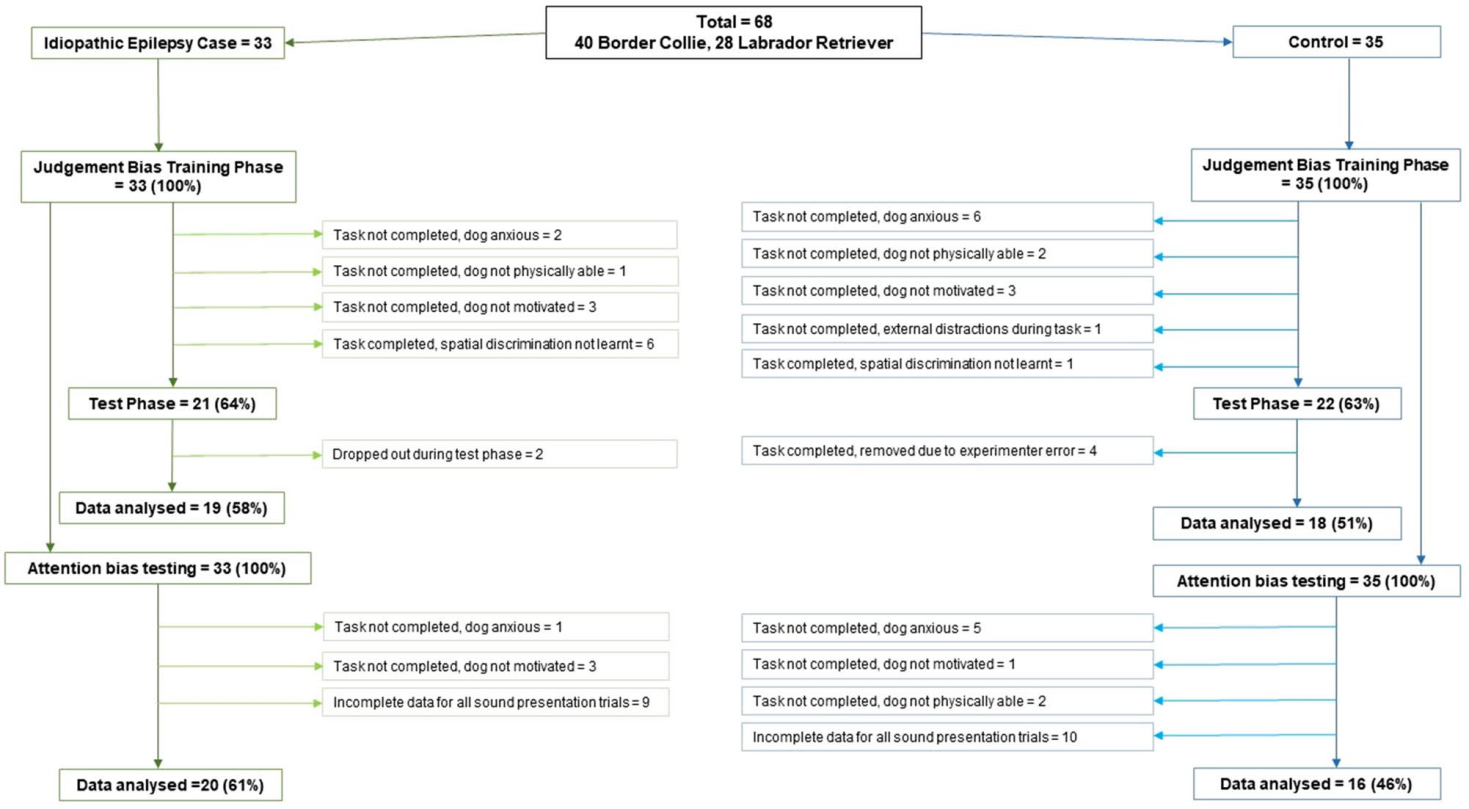
Schematic of the population and sub-population of dogs recruited to the study, and number of dogs included in analyses for each behavioural task. Percentage of dogs remaining in the study at are reported for each stage of the study by study group.

Of the 68 dogs that took part in the behavioural tasks, n = 37 met criterion and completed the judgement bias task including the test phase (n = 19 IE, n = 18 Control). Reasons for non-completion of this task are outlined in Fig. 2 and included lack of acquisition of the spatial discrimination (and thus probes not tested), and several dog-related factors that led to the training phase not being completed e.g. lack of motivation, anxiety, and physical inability (e.g. lethargy, ataxia) inhibiting performance. There were significant differences in reasons why the judgement bias testing stage was not reached between groups, with the IE group more likely due to cognitive reasons (i.e. completing the task but not reaching criterion) (IE = 50%, Control = 8%), while the control group was more likely due to behavioural and physical reasons (i.e. being unable to complete the task due to anxiety or lack of motivation) (IE = 50%, Control = 92%) (*X*^2^ = 5.54, p = 0.019). Within the sub-population with IE, there was no effect of ASD treatment on likelihood of reaching the testing stage (ASD-treated: testing phase reached = 54.5% vs. Drug-naïve: 63.6%; *X*^*2*^ = 0.25, p = 0.618).

Dogs that reached criterion during the training phase achieved this in a median of 32 trials (IQR 21–42). IE diagnosis did not affect learning speed (Control: median 33 (IQR 24–46.5); IE: median 30 (IQR 20–37); U = 126.5, z = − 1.36, p = 0.18). Within the sub-population with IE, there was no effect of ASD treatment on trials to criterion (ASD-treated: median 34.0 (IQR 20.0–38.5); Drug-naïve: median 30.0 (IQR 23.0–37.0); U = 36.0, z = 0.35, p = 0.78).

**Figure 3:**
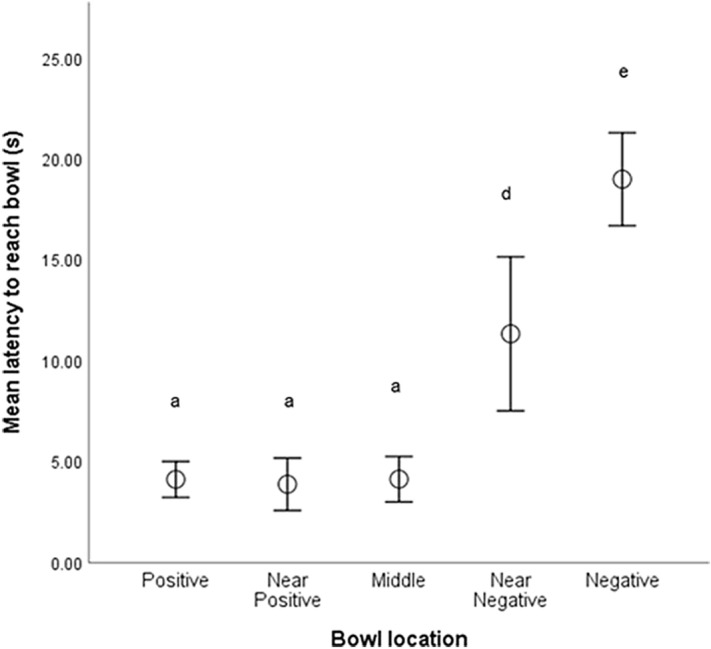
Mean latencies (± SE) for dogs to reach each bowl location in the judgement bias test. Pairwise Dunn-Bonferroni test significant differences (p < 0.05) were found between locations that do not share any of the same letter superscripts.

Type of goal location affected latency (Friedman’s test: *X*^*2*^ = 84.46, df = 4, n = 37, p < 0.001) with dogs reaching the P location fastest and N location slowest, and intermediate latencies for ambiguous bowl locations (NP, MID, NN) indicating that they had learnt the task (Fig. 3). IE and Control dogs did not differ in their unadjusted latencies to P and N locations (P: U = 191.5, z = 0.62, p = 0.53; N: U = 172.0, z = 0.30, p = 0.98).

To compare responses of IE and control dogs to the ambiguous probe locations in the test phase, unadjusted latency data were first used in a linear mixed model (LMM). Dogs responded differently between the three ambiguous locations (LMM, F_2,102_ = 11.8, P < 0.001). There was no main effect of study group (LMM, F_1,102_ = 0.20, p = 0.658), or interaction between study group and bowl location (LMM, F_2,102_ = 0.11, p = 0.900). We found no sex, age or breed differences for latencies (LMM, sex: F_1,102_ = 0.41, p = 0.521; age: F_1,102_ = 0.031, p = 0.861; breed: F_1,102_ = 1.21, p = 0.274). This model was repeated with adjusted latencies as the outcome measures, and the same effects were found with type of goal location significant (LMM, F_2, 102_ = 14.80, P < 0.001), but no effect of study group (LMM, F_2, 102_ = 0.01, P = 0.934), interaction between study group and bowl location (LMM, F_2, 102_ = 0.152, P = 0.859), breed (LMM, F_2, 102_ = 1.17, P = 0.291), sex (LMM, F_2, 102_ = 0.499, P = 0.482) or age (LMM, F_2, 102_ = 0.75, P = 0.388).

### Attention bias

**Figure 4:**
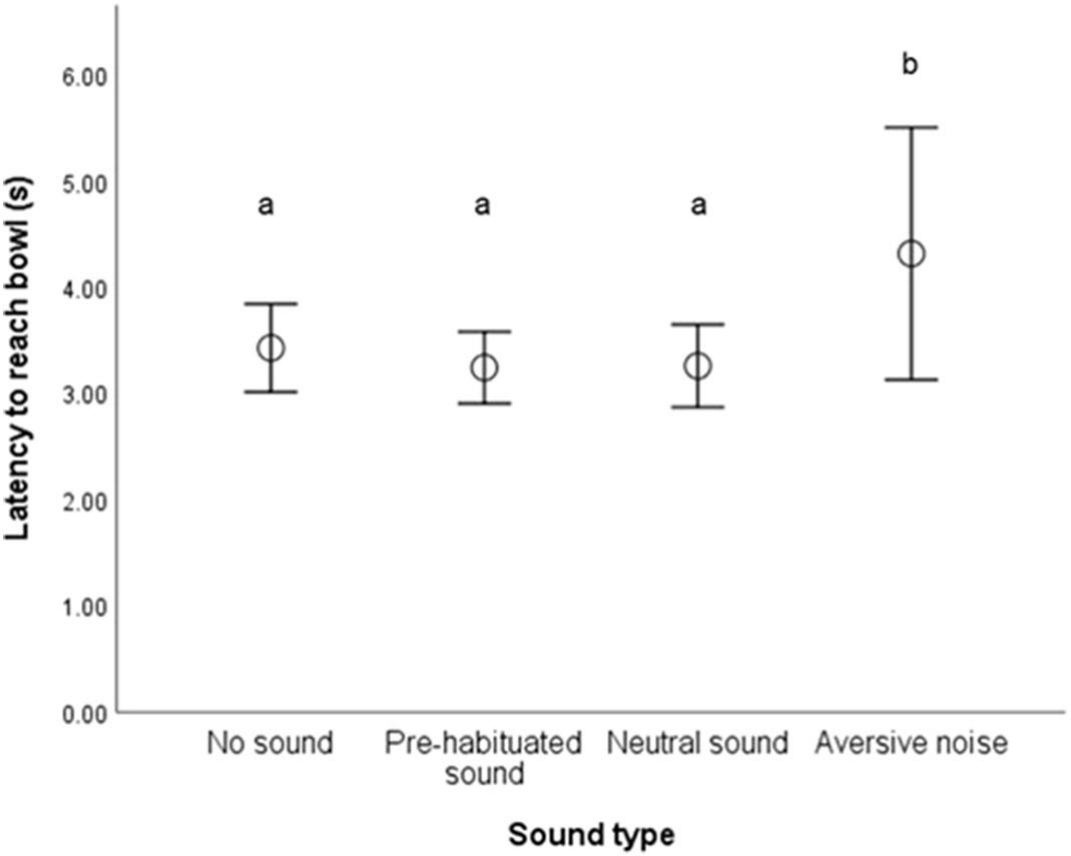
Mean latencies (± SE) for dogs to reach the baited bowl following different sound types being played in the attention bias test. Pairwise Dunn-Bonferroni test significant differences (p < 0.05) were found between sounds that do not share any of the same letter superscripts.

Of the 68 dogs that took part in the behavioural tasks, n = 56 took part in the attention bias task (IE n = 29, Control n = 26). Of this population, 36 dogs completed the full task and data from trials of all sounds were analysed (n = 20 IE, n = 16 Control). In an LMM with latency to P as the outcome measure, there was a significant effect of sound type (LMM, F_3,102_ = 4.35, p = 0.006) (Fig. 4). Dogs reached P significantly slower following exposure to an aversive sound compared to no, neutral or pre-habituated sounds There was no effect of breed (LMM, F_1,31_ = 0.519, p = 0.477) or sex (LMM, F_1,31_ = 0.462, p = 0.502) on latency to reach P. Age had a significant positive effect on latency (LMM, F_1,31_ = 5.09, p = 0.031), with longer latencies in older dogs. There was no interaction between study group (IE vs. Control) and sound type (LMM, F_3,102_ = 1.64, p = 0.184), nor a main effect of study group on latency (LMM, F_1,31_ = 0.659, P = 0.423).

When considering the relationship between dogs’ responses in the attention and judgement bias tasks (n = 26 for which data were available for both tasks), a positive correlation was found between latency to reach the bowl in response to the aversive sound (where higher latency indicates an increased attention to threat) and latency to reach the NP probe location (where higher latency indicates a less optimistic response) (r_s_ = 0.569, n = 26, p = 0.002); however, no correlation was found with other probe locations (MID: r_s_ = 0.356, n = 26, p = 0.074); NN: r_s_ = 0.241, n = 26, p = 0.236).

## Discussion

Idiopathic epilepsy did not induce a negative judgement or attention bias in our sample of dogs compared to healthy controls, when a commonly used judgement bias test and a novel auditory attention bias test was applied. This suggests that in this study population, epilepsy does not induce a negative long-term affective state (i.e. mood^53^). However, epilepsy could nonetheless induce negative short-term affective states (e.g. immediately post-ictal negative emotions), and in light of previous evidence demonstrating negative effects of epilepsy upon owner-reported quality of life (not limited to the post-ictal period)^54^, chronic changes to stress physiology indicative of hypothalamic-pituitary adrenal axis dysregulation^55^ and co-morbid affective disorders in the inter-ictal period (e.g. anxiety)^18^, this is an unexpected finding. These negative results may be due to a number of factors, which will now be discussed.

A key issue in this study was the attrition of the study sample from the start to the end of testing, and thus the quantity of useable data and the characteristics of the resulting sample analysed. A major reason for loss of data in the judgement bias task was dogs with IE being unable to learn the discrimination within the allotted trials. Dogs with IE were more likely to lack probe data due to not meeting criterion in the training phase, compared to controls. Over one fifth (6/27; 22.2%) of dogs with IE that completed the training phase did not meet learning criterion compared to just 1/23 (4.3%) of controls. This may reflect the cognitive impairments recently identified in dogs with IE^14–16^, particularly in spatial working memory^16^. In contrast, in a recent study of 125 dogs trained on the same spatial discrimination, only one dog (0.8%) did not reach the same learning criterion after completing the training phase^56^. Negative effects of epilepsy upon cognition are well documented in people with epilepsy, ranging from mild impairments to severe cognitive decline^57^. In addition to the effects of epilepsy and seizures themselves, cognitive side effects of ASDs are reported in people^58^. ASD treatment may have impaired cognition and led to a lack of probe data for some dogs in this study population. Ideally, all IE dogs would have been tested while drug-naïve to test the effects of epilepsy alone; however, for ethical reasons it was not possible to withhold medications. As dogs with IE were treated with varying combinations of medications, and none of these drugs were used to treat dogs in the control group, statistically assessing their individual effects in the overall population was not possible. However, comparison of ASD-treated vs. drug-naïve dogs in the IE sub-population indicated that ASD treatment did not affect learning speed in the judgement bias task, or likelihood to reach the judgement bias testing stage. Despite this, it is possible that within the ASD-treated group some combinations of therapies may have been particularly cognitively inhibiting; for example, all four dogs receiving three ASDs as polytherapy (the highest number of ASDs in this population) did not complete the cognitive bias task and thus their probe data were unavailable. Dogs with IE treated with polytherapy have previously been found to exhibit more significant cognitive impairments than those treated with one ASD or drug-naïve dogs^14^. Designing tests to measure affective state that avoids intensive training and could be used in cognitively impaired individuals remains a challenge in animal welfare science.

A further reason for loss of data was some dogs being too anxious to perform in the tasks, leading to the experimenters electing to cease testing. For example, some dogs chose to leave the testing arena and instead approach the exit door on commencement of each trial. As dogs exhibiting anxious behaviour in the testing context would be valuable to study in cognitive bias paradigms (given they are likely to have an overall more negative affective state than individuals not exhibiting these behaviours), methods by which affective state can be assessed in this challenging population should be developed. Barnard et al.^59^ previously suggested the use of attention bias tasks instead of judgement bias tasks to reduce the level of stress associated with testing, and avoid the need for extensive training. Although not discriminatory between study groups, the novel auditory attention bias task described here shows promise, with dogs taking significantly longer to reach the rewarded location when exposed to a threatening auditory cue compared with neutral or pre-habituated auditory cues. This may be due to their attention being oriented away from the reward and towards the threatening cue, due to its increased salience compared with non-threatening or familiar sounds; however, further detailed behavioural analysis (e.g. changes in ear and head position) and physiological measures (e.g. heart rate) are needed to further support this. This paradigm allowed some dogs unable to participate in the full training for the cognitive bias task to be tested and used a biologically relevant stimuli to trigger innate emotional responses. Such approaches are increasingly being explored in farm^42,44,46,60,61^ and laboratory^62^ animals, but have been relatively neglected in companion animals to date. Further refinement of the task presented here is needed; some dogs still failed to complete attention bias task as well as the judgement bias task due to anxiety. It is likely that further adaptation of this task, including performing tests in the dog’s home environment instead of an unfamiliar testing arena following car travel would be preferable, dependent upon whether sound levels could be adequately controlled in the home. In addition, as this study tested the unknown affect treatment of epilepsy in a novel attention bias task, further validation of this task is required, including exploring the responses of dogs with known affect treatments e.g. syringomyelia in Cavalier King Charles Spaniels^37^, or separation related behaviour^38^, both of which have been found to induce a negative affective state in dogs, with this paradigm.

It is possible that the population of dogs with IE sampled here did not experience sufficiently severe epilepsy or anxiety to induce perturbations in affect severe enough be detected in the paradigms tested. The selection process for breeds for this study was based upon their popularity, epilepsy prevalence and severity. Epilepsy in Border Collies is frequently characterised by a severe clinical course and poor response to medical treatment^64^. In previous studies, 71% of Border Collies were considered drug-resistant on two or more ASDs^64^, with as few as 0–18%^48,64^ of dogs achieving remission (seizure freedom). Although dogs in this sample had some severe clinical features (e.g. high prevalence of cluster seizures), the majority (85.0%) appeared drug-responsive and had achieved a ≥ 50% reduction in seizure frequency following ASD treatment (for drugs treated with monotherapy) or the addition of their most recent ASD (for dogs treated with polytherapy). ASD resistance has been linked to behavioural co-morbidities; in rodent models of epilepsy, rats with greater drug-resistance show greater neurobehavioural abnormalities^65^, and human epilepsy patients with poorer seizure control have a higher likelihood of mood disorders^66^. As such, sampling more drug-resistant dogs with higher seizure frequencies and behavioural comorbidities, or dogs with stress induced seizures^11^ may have increased the likelihood of detecting changes in affective state. Accessing such populations for practical studies may be challenging. Dogs recruited to this study are likely to have exhibited less severe epilepsy and anxiety due to self-selection of owners volunteering to take part in the study. Owners of dogs with severe or uncontrolled epilepsy may be less likely to engage in voluntary activities that involve stressors such as car travel, novel environments and meeting strangers through fear of stress triggering a seizure in their dog^11,63^. This was mitigated where possible by testing in a non-clinical environment, habituating dogs to the experimenters and testing room, and using positive training techniques, which was communicated to owners; however, many may have still avoided such activities. The same may apply for dogs with more profound anxiety; owners of dogs with IE with co-morbid anxiety may have been more cautious in signing up their dog than owners of control dogs exhibiting anxiety, thus minimising differences between the study groups. Indeed, during the sign-up process, the authors noted that more owners from the IE group requested additional details regarding the tasks and potential stressors than control owners. In addition, on registering interest in the study, owners of dogs with problems with aggression, particularly around strangers and food, were screened out of the study. It has previously been highlighted that screening out of dogs with more extreme behaviour is typical with cognitive bias research due to concerns for experimenter safety and welfare of the dog^59^. As such it is probable that extremely anxious dogs were not represented in this sample, particularly not in the IE group.

Both ASDs and some dietary supplements have potential psychoactive effects that may have improved underlying affective state (e.g. acting as an anxiolytic to co-morbid anxiety) in the IE group, obscuring potential differences with controls. The majority of dogs with IE (22/33) were treated with ASDs, with twelve receiving polytherapy (two or more ASDs). Imepitoin has been found to have anxiolytic as well as antiseizure effects in physically healthy dogs^67–69^; however, no changes in anxiety levels were reported by owners of dogs treated with imepitoin for IE^70^. In addition, several dogs were supplemented with various oils as adjunctive treatments, including MCT and CBD oil, a practice increasingly common in this population^71^. In a previous randomised placebo-controlled crossover trial of an MCT-enriched diet, dogs with epilepsy were found to have reduced stranger-directed fear during the MCT phase than the placebo phase^19^, with anxiolytic effects of MCTs also seen in rodents^72^. Although there is increasing interest in the use of CBD for mood disorders, with some evidence of anxiolytic effects in humans^73–75^, effects in dogs are unknown.

The timing of testing may further explain the lack of effect identified in this study. Dogs with IE were tested at least 24 h after their last seizure and were thus in the ‘inter-ictal’ (between seizure) state. The post-ictal state that immediately follows a seizure is commonly characterised by distress, pacing, lethargy, polyphagia and ataxia^10^, and thus may conceivably negatively influence a dog’s mood. Dogs were not tested during this state due to both practical and welfare considerations. It is possible that during the inter-ictal phase, dogs who are otherwise unaffected by side effects of ASDs and/or behavioural comorbidities of epilepsy do not differ in their affective state from healthy control dogs.

Due to the heterogeneity of the case population, it is possible our sample may have provided insufficient power to detect an effect of epilepsy. This is less likely, given our sample of over 35 dogs for each task (judgement bias n = 37; attention bias, n = 36), which is higher than other comparable studies that detected differences in affective state using the judgement bias paradigm e.g. eight dogs with syringomyelia vs. 13 controls^37^. However, as epilepsy is a heterogenous disease, even within-breed, it is possible that a larger sample is needed.

Responses to the ambiguous stimuli in the judgement bias task differed from some other studies. Rather than an intermediate response to the middle bowl (as seen in Cockburn et al.^37^ and others), dogs showed similar response latencies to NP and MID, with a significant increase in latency to NN. Such response patterns were previously described in Muller et al.^76^, and were speculated to be attributable to personality differences. Individual variability in response to the probes requires further investigation given it may cause inconsistencies in cognitive bias results.

## Conclusions

Our study found that idiopathic epilepsy had no detectable effect on long-term judgement or attention bias in this particular study population when compared to matched controls; however, these results require further investigation due to the attrition of dogs with greater situational anxiety (in both case and control groups) and cognitive impairment (particularly in dogs with idiopathic epilepsy) from the study sample, and the relatively mild phenotype of the idiopathic epilepsy cases. Our results provide further evidence for the recently recognised cognitive impairments in canine epilepsy patients, that are well documented in human epilepsy patients. It is important that future tests of affective state are designed to allow accurate assessments of animals who are unable to perform in anxiogenic environments or cognitively perform in extensive testing. The novel auditory attention bias task we present here allowed more dogs to perform than the validated judgement bias task, potentially due to the reduced level of training required; however, further refinements are needed, for example, conducting the task in a familiar testing environment such as the dog’s home.

## Supplementary information

Supplementary Information.
