# Supplementary material for: Impact of canine epilepsy on judgement and attention biases
Source: Sci Rep. 2020 Oct 20;10:17719. doi: 10.1038/s41598-020-74777-4 (PMC7576193; doi:10.1038/s41598-020-74777-4)
Supplement: Supplementary file 1 — Supplementary Information. [file 41598_2020_74777_MOESM1_ESM.docx]

**Impact of canine epilepsy on judgement and attention biases**

Sarah L Hobbs^1^, Tsz Hong Law^1^, Holger A Volk^1,2^, Chantal Younis^1^, Rachel A Casey^3^, Rowena MA Packer^1*^

^1^ Royal Veterinary College, Hawkshead Lane, Hatfield, Hertfordshire, AL9 7TA, UK

^2^ Department of Small Animal Medicine and Surgery, University of Veterinary Medicine Hannover, Bünteweg, 30559 Hannover, Germany

^3^ Dogs Trust, 17 Wakley Street, The Angel, London, EC1V 7RQ, UK

**Supplementary Table 1 Signs of stress, anxiety and frustration each study dog was monitored for before, during and after behavioural tasks** (adapted from <http://www.bristol.ac.uk/vetscience/services/behaviour-clinic/dogbehaviouralsigns/>)

| **Signs of fear/anxiety** | **Responses to sound** | **Sensitisation** |
| --- | --- | --- |
| Yawning repeatedly, excessive salivation, panting, lip licking repeatedly (not in response to food) | Ears pricking/move backwards briefly | Continued vocalisation in response to sound |
| Vocalisation: whining, howling, barking, whimpering | Looking around briefly | Startle response |
| Tense body, or face. Tail tucked, low body posture, freezing | Looking towards sound briefly | Hiding/running away from sound |
| Wide eyes, enlarged pupils, showing whites of eyes | Head tilt | Continuous pacing or not able to settle |
| Ears held back and low (unless normal for dog) | Moves towards sound | Salivation |
| Appeasement grin, or roll | Interrupts other activity | Repetitive behaviour |
| Avoidance (including avoidance of interaction or eye contact, physically moving away, reluctant to engage with instructions, approaching and retreating repeatedly, hiding) | Sniffs floor/self in response to sound | Increased interaction with people e.g. attention seeking |
| Any form of aggression (including lip raising, showing teeth, snarling, growling) | Vocalise | Continued or increase of other listed behaviours |
| Hackles raised | Shaking off |  |
| Pacing, lunging, shaking off, trembling, paw raise, startling more easily | Yawning |  |
| Excessive attention seeking (including jumping up, mouthing) |  |  |
| Not eating |  |  |
| Repetitive behaviour |  |  |
| Toileting (in response to stimuli) |  |  |
